# Automatic construction of metabolic models with enzyme constraints

**DOI:** 10.1186/s12859-019-3329-9

**Published:** 2020-01-14

**Authors:** Pavlos Stephanos Bekiaris, Steffen Klamt

**Affiliations:** Max Planck Institute for Dynamics of Complex Technical Systems, Sandtorstr. 1, Magdeburg, Germany

**Keywords:** Flux balance analysis, *Escherichia coli*, Metabolic modeling, Enzyme constraints, Protein allocation, Minimal cut sets, Proteomics

## Abstract

**Background:**

In order to improve the accuracy of constraint-based metabolic models, several approaches have been developed which intend to integrate additional biological information. Two of these methods, MOMENT and GECKO, incorporate enzymatic (kcat) parameters and enzyme mass constraints to further constrain the space of feasible metabolic flux distributions. While both methods have been proven to deliver useful extensions of metabolic models, they may considerably increase size and complexity of the models and there is currently no tool available to fully automate generation and calibration of such enzyme-constrained models from given stoichiometric models.

**Results:**

In this work we present three major developments. We first conceived short MOMENT (sMOMENT), a simplified version of the MOMENT approach, which yields the same predictions as MOMENT but requires significantly fewer variables and enables direct inclusion of the relevant enzyme constraints in the standard representation of a constraint-based model. When measurements of enzyme concentrations are available, these can be included as well leading in the extreme case, where all enzyme concentrations are known, to a model representation that is analogous to the GECKO approach. Second, we developed the AutoPACMEN toolbox which allows an almost fully automated creation of sMOMENT-enhanced stoichiometric metabolic models. In particular, this includes the automatic read-out and processing of relevant enzymatic data from different databases and the reconfiguration of the stoichiometric model with embedded enzymatic constraints. Additionally, tools have been developed to adjust (kcat and enzyme pool) parameters of sMOMENT models based on given flux data. We finally applied the new sMOMENT approach and the AutoPACMEN toolbox to generate an enzyme-constrained version of the *E. coli* genome-scale model *i*JO1366 and analyze its key properties and differences with the standard model. In particular, we show that the enzyme constraints improve flux predictions (e.g., explaining overflow metabolism and other metabolic switches) and demonstrate, for the first time, that these constraints can markedly change the spectrum of metabolic engineering strategies for different target products.

**Conclusions:**

The methodological and tool developments presented herein pave the way for a simplified and routine construction and analysis of enzyme-constrained metabolic models.

## Background

Constraint-based metabolic models (CBM) have become a powerful framework for describing, analyzing, and redesigning the cellular metabolism of diverse organisms (see reviews [1–3]). A minimum constraint-based model consists of the stoichiometric matrix of the metabolic network under study, the reversibility of the reactions and some upper or lower flux bounds, typically of exchange reactions. Assuming a steady state of the internal metabolite concentrations, a mass balance equation is formulated using the stoichiometric matrix. This equation, together with the flux bounds, defines a space of feasible flux distributions in the metabolic network which is then analyzed by various methods [1–3], including flux balance analysis (FBA, see review [4]), metabolic pathway analysis [5, 6] or computational strain design [7]. While the mass balances represent the most important constraint, various extensions of CBM have been proposed which incorporate additional biological data with the goal to further constrain the solution space and thus to improve the accuracy of predictions. This includes the integration of different omics [8] and thermodynamic data [9]. One particular branch of these methods deals with the inclusion of enzyme constraints which basically rely on the fact that there is a limited amount of protein in a cell which needs to be optimally allocated to the different (in particular metabolic) processes. This naturally raises an optimization problem of optimal enzyme allocation and it has been shown that the incorporation of these constraints in CBM indeed leads to better predictions, for example, of overflow metabolisms and of the Crabtree effect [10, 11] as well as of growth rates without explicitly limiting the substrate uptake rates [12, 13]. Over the last years, quite a number of different (but often related) approaches for CBM with protein allocation constraints have been proposed ranging from the inclusion of enzyme requirements in metabolic reactions (e.g., FBA with molecular crowding (FBAwMC, [12]) and its extensions MOMENT [13] and GECKO [11]) up to the very detailed description of the synthesis of proteins (and of other cellular components) including resource balance analysis (RBA, [14, 15] and Metabolism-Expression models (ME models [16]). While such fine-grained models allow, for example, the explicit inclusion of transcription and translation processes, they also require much more biological data (e.g. translation and transcription efficiencies) in order to obtain valid model predictions. For many organisms, such data are not available. In contrast, simpler approaches such as MOMENT and GECKO basically need as input the molecular weight as well as the (maximal) turnover number k_cat_ (or, alternatively, the apparent or effective turnover number k_app_) of the involved metabolic enzymes. This information is readily available for many (organism-specific) enzymes in databases such as SABIO-RK [17] or BRENDA [18]. MOMENT was applied on the genome-scale *E. coli* model *i*JO1366 [19]. Without restricting maximal carbon source uptake rates, this MOMENT-applied model successfully showed superior aerobic growth rate predictions for 24 different carbon sources compared to the original *i*JO1366, thus explaining the growth rates with enzyme mass constraints only. GECKO (Genome-scale model enhancement with Enzymatic Constraints accounting for Kinetic and Omics data [11]) uses the same type of protein allocation constraints but in a more explicit manner. It introduces additional reactions and metabolites to reflect enzyme usage. As the main advantage, this representation allows the direct incorporation of measured enzyme concentrations implying upper limits for flux capacities. GECKO was successfully used for a *Saccharomyces cerevisiae* model together with in vivo proteomic data. In particular, this GECKO model exhibited the Crabtree effect [20], i.e. the switch to fermentative metabolism in yeast at high glucose uptake rates, without explicitly bounding substrate or oxygen uptake rates.

The present work has three major goals. First, we introduce the sMOMENT (short MOMENT) method for the inclusion of protein allocation constraints in stoichiometric metabolic models. It is primarily based on MOMENT, but, due to simplifications, it requires considerably less variables and the resulting constraints can directly be incorporated in the stoichiometric matrix. This not only reduces the computational demand for complex calculations (e.g., determination of minimal cut sets [21]) but also facilitates the direct application of standard software tools for constraint-based modeling for the analysis of sMOMENT models. We also show how protein concentration measurements can be integrated in sMOMENT models mimicking the functionality of GECKO models, but again needing much smaller models (as long as concentration measurements are only available for a subset of all enzymes). Second, we present the AutoPACMEN (Automatic integration of Protein Allocation Constraints in MEtabolic Networks) toolbox allowing an almost fully automated creation of sMOMENT metabolic models. In particular, this includes the automatic read-out of the relevant enzymatic data from the SABIO-RK [17] and BRENDA [18] (and optional custom) databases and the reconfiguration of the stoichiometric model to embed the enzymatic constraints according to sMOMENT. AutoPACMEN can be used for any organism and stoichiometric model and requires only the SBML representation of the metabolic model as primary input. Additionally, AutoPACMEN provides tools to adjust parameters of sMOMENT models based on experimental flux data.

Finally, as an exemplary use of the new AutoPACMEN toolbox and as illustration of the sMOMENT method, we applied AutoPACMEN to generate an sMOMENT-enhanced version of the *E. coli* genome-scale model *i*JO1366. We then compare the original model with the sMOMENT model with respect to various properties. In particular, we show that the sMOMENT model significantly improves flux predictions (including overflow metabolism) and we demonstrate, for the first time, that enzyme constraints may significantly change the spectrum of metabolic engineering strategies.

## Methods

### The sMOMENT method

We assume that we are given a constraint-based metabolic model in standard form with its stoichiometric matrix ***S*** and flux vector ***v*** together with steady state mass balances

1 $$ \boldsymbol{Sv}=\mathbf{0} $$

and upper and lower bounds for the fluxes

2 $$ {\alpha}_i\le {v}_i\le {\beta}_i. $$

We further assume that, in a preprocessing step, reversible reactions of enzymatically catalyzed reactions in the metabolic network model are split into two irreversible (forward and backward) reactions (with *α*_*i*_ ≥ 0).

In order to incorporate adequate enzyme (mass) constraints in a given metabolic model, MOMENT [13] first introduces, for each enzyme-catalyzed reaction *i*, an enzyme concentration variable *g*_*i*_ (mmol/gDW). We initially assume that a reaction is catalyzed by a unique enzyme. The flux *v*_*i*_ (mmol/gDW/h) through reaction *i* is then limited by the product of the enzyme concentration and the (maximal) turnover number, *k*_*cat*,*i*_ (1/h), of this enzyme:

3 $$ {v}_i\le {k}_{cat,i}\bullet {g}_i $$

which can alternatively be written as

4 $$ \frac{v_i}{k_{cat,i}}\le {g}_i. $$

(Note that the *k*_*cat*,*i*_ values may differ for forward and backward direction of (split) reversible reactions). In order to reflect the limited amount of metabolic enzymes in the cell another constraint is introduced stating that the sum of all enzymes in the model may not exceed a threshold *P* (g/gDW):

5 $$ \sum {g}_i\bullet {MW}_i\le P. $$

*MW*_*i*_ is the molecular weight (g/mmol) of the enzyme catalyzing reaction *i*. It should be noted that *P* only refers to metabolic enzymes (covered by the metabolic model) and is thus smaller than the total protein content of the cell.

When applying MOMENT to a genome-scale model, a great number of additional variables *g*_*i*_ and their associated constraints (4) must be introduced which may negatively affect the performance of complex analyses of the resulting model. Furthermore, the constraints (4) and (5) cannot be directly integrated into the standard form of a metabolic model represented by (1) and (2). For this reason, MOMENT models cannot be directly treated with standard tools for constraint-based modeling (such as [22–24]). In order to tackle these issues, we developed the sMOMENT (short MOMENT) method which leads to the same results as MOMENT but uses a more compact representation of the model. Using (4) we first substitute *g*_*i*_ in (5) and obtain:

6 $$ \sum {v}_i\bullet \frac{MW_i}{k_{cat,i}}\le \sum {g}_i\bullet {MW}_i\le P. $$

We can thus safely use the following alternative for (5):

7 $$ \sum {v}_i\bullet \frac{MW_i}{k_{cat,i}}\le P. $$

This inequality can be reformulated as follows:

8 $$ -\sum {v}_i\bullet \frac{MW_i}{k_{cat,i}}+{v}_{Pool}=0;{v}_{Pool}\le P. $$

**Fig. 1:**
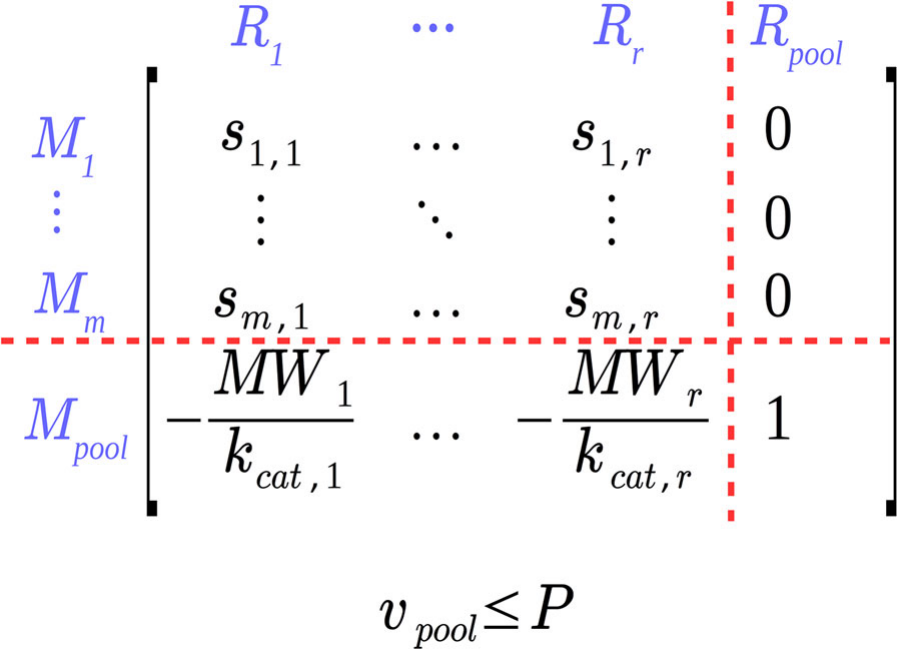
Augmentation of the stoichiometric matrix with the sMOMENT approach. *M*_*pool*_ is the enzyme pool pseudo-metabolite and *R*_*pool*_ the enzyme-pool-delivering pseudo-reaction. *R*_*i*_ stands for reaction *i*, *M*_*j*_ for metabolite *j*; *r* is the number of reactions, *m* the number of metabolites

The auxiliary variable *v*_*Pool*_ (g/gDW) quantifies the mass of all metabolic enzymes per gram of cell dry weight needed to catalyze the reaction fluxes *v*_*i*_ and this value must not exceed the given maximum *P*. The advantage of (8) is that it can directly be integrated in the standard system defined by (1) and (2) (Fig. 1). First, a pseudo-metabolite (enzyme pool) is added as a new row in the stoichiometric matrix where the stoichiometric coefficient for each reaction *i* is $\left(-\frac{MW_i}{k_{cat,i}}\right)$. Afterwards, a pseudo-reaction *R*_*pool*_ (“enzyme delivery”) is added whose coefficients in ***S*** are all zero except unity for the added enzyme pool pseudo-metabolite and the associated “enzyme delivery flux” *v*_*Pool*_ has an upper bound of *P* (Fig. 1).

The integration of the enzyme mass constraints in the stoichiometric matrix as shown in Fig. 1 is similar to the one used by GECKO [11] but it markedly differs from it as it avoids explicit introduction of enzyme species and their delivery reactions which largely increases the dimension of GECKO models. To achieve that, special treatment is needed for reactions catalyzed by multiple enzymes as well as for multifunctional (promiscuous) enzymes. The handling of these cases in sMOMENT is similar to MOMENT but again simplified compared to MOMENT’s usage of recursive rules. Herein we consider an enzyme as an entity that can catalyze one or, in the case of multifunctional enzymes, several reactions. An enzyme can be either a single protein or an enzyme complex consisting of multiple proteins. Genome-scale metabolic models often provide gene-enzyme-reaction relationships which are essential to build enzyme-constrained metabolic models because they enable one to associate reactions with their catalyzing enzymes as well as enzymes with the respective genes and gene products needed to build that enzyme (or enzyme complex). We denote by *E* the set of all *q* enzymes of a metabolic model:

9 $$ E=\left\{{E}^1,\dots, {E}^q\right\}. $$

Every enzyme *E*^*j*^ has its own molecular weight ${MW}_{E^j}$ (g/mmol) which can be directly derived from the masses of its amino acids (in the case of enzyme complexes, its molecular weight is the sum of the single protein masses, each multiplied with the stoichiometry of the single protein in the complex). This information is readily available in databases such as UniProt [25]. Additionally, each enzyme *E*^*j*^ has an associated k_cat_ value ${k}_{cat,{E}^j}$. With *E*(*i*) we denote the enzyme(s) that catalyze reaction *i*:

10 $$ E(i)=\left\{{E}^{i1},{E}^{i2},\dots \right\} $$

For setting the enzyme costs *c*_*i*_ = *MW*_*i*_/*k*_*cat*,*i*_ of reaction *i* in the eqs. (5)–(8) sMOMENT selects the minimal enzyme costs of all enzymes catalyzing reaction *i*:

11 $$ {c}_i=\frac{MW_i}{k_{cat,i}}=\min \left(\left\{\frac{MW_{E^{i1}}}{k_{cat,{E}^{i1}}},\frac{MW_{E^{i2}}}{k_{cat,{E}^{i2}}},\dots \right\}\right);{E}^{i1},{E}^{i2},\dots \in E(i). $$

This rule used by sMOMENT simplifies the treatment of reactions with multiple enzymes but does not change the feasible flux space because the solution with minimal protein costs used by sMOMENT is contained in the corresponding MOMENT or GECKO model as well (and will in fact be selected in these models by the solver in optimizations where the protein pool becomes limiting). While the flux space of sMOMENT and predictions made therein are thus identical to MOMENT and GECKO, the latter two hold explicit variables for the involvement of each enzyme and can thus account for all possible enzyme combinations that can generate a given flux in the case where a reaction can be catalyzed by multiple enzymes (whereas sMOMENT always assumes that the enzyme with the minimal cost is used). However, this additional information is rarely relevant and in cases where the solutions of the optimization is limited by the protein pool, the enzyme with the minimal enzyme costs (as favored by sMOMENT) will be selected. If a reaction has no associated enzyme we set the term $\frac{MW_i}{k_{cat,i}}$ (and thus the enzyme costs) in eq. (8) to 0.

As already stated above, GECKO [11] was introduced as an extension of MOMENT. It uses the same type of enzyme mass constraints but introduces additional reactions and metabolites to explicitly reflect enzyme usage. The disadvantage is that the model size increases significantly which hampers its use in computationally expensive analyses. On the other hand, this representation allows the direct incorporation of measured in vivo proteomic concentrations as upper limits for enzyme usage. Equivalently to GECKO, although not further used herein, it is possible to include proteomic concentration data in the sMOMENT method as well. Assuming we are given the concentration [*E*^*k*^] of an enzyme *E*^*k*^ (mmol/gDW) and that this enzyme is the only catalyst in the reaction(s) it catalyzes, this immediately sets an upper bound for the sum of all reaction fluxes catalyzed by enzyme *E*^*k*^:

12 $$ \sum \limits_{i\epsilon R\left({E}^k\right)}\frac{v_i}{k_{cat,i}}\le \left[{E}^k\right] $$

where *R*(*E*^*k*^) denotes the set of reactions catalyzed by enzyme *E*^*k*^. Similar as we did for the overall protein pool (cf. eq. (7) and (8)) we may include this constraint by adding an additional pseudo metabolite and pseudo reaction in the stoichiometric matrix.

For the case that *E*^*k*^ is not the only catalyzing enzyme in a reaction *i* it catalyzes, we split this reaction in two reactions with the same stoichiometry, one reaction is now (exclusively) catalyzed by enzyme *E*^*k*^ while the other reaction is catalyzed by all other enzymes of the former reaction *i* (i.e., *E*(*i*)\*E*^*k*^). Thereby, the rule (11) has to be applied again for both of the new reactions and the respective (possibly adapted) enzyme cost values have to be used in eq. (8) and in the augmented stoichiometric matrix. In case that the split reaction *i* had a limited flux bound (*v*_*i*_ < ∞), additional constraints must be introduced (e.g. “arm” reactions as used in the GECKO approach) to ensure that this constraint is met by the sum of all the reactions obtained by splitting reaction *i*.

The procedure outlined above has to be repeated for all enzymes with measured concentrations. With a growing set of concentration measurements, this will add several new columns and reactions in the stoichiometric matrix. However, concentration measurements are often available only for a small fraction of all enzymes. In these cases, the size of the augmented sMOMENT model as described above will still be significantly smaller than a fully expanded GECKO model. If concentrations are specified for all enzymes then the resulting model will, in fact, be an analogon to a GECKO model with the same number of reactions and metabolites. In principle, when using the AutoPACMEN toolbox (see below), very high (non-limiting) concentrations can be defined during model generation to enforce explicit inclusion of some or of all enzymes (in the latter case, a GECKO-analogous model will be generated); these concentration values can later be adapted for a given set of measurements.

### AutoPACMEN toolbox

**Fig. 2:**
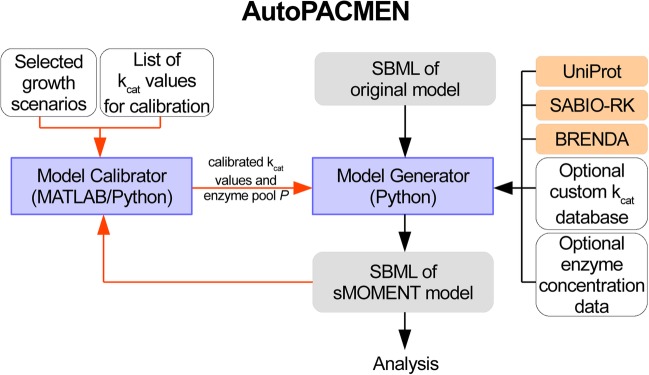
General overview of the structure and workflow of the AutoPACMEN toolbox consisting of the model generator and model calibrator. The red arrows show the optional model calibrator workflow. The blue boxes indicate AutoPACMEN programs, the grey boxes for input and output files of AutoPACMEN, the orange boxes for external databases which are read out by the AutoPACMEN programs, and white boxes for optional datasets which can be provided by the user

The AutoPACMEN (Automatic integration of Protein Allocation Constraints in Metabolic Networks) toolbox implements the sMOMENT method. It consists of two parts (Fig. 2): (1) the AutoPACMEN model generator for the automatic generation of an sMOMENT-enhanced version of a stoichiometric metabolic model, and (2) the AutoPACMEN model calibrator which helps fitting parameters of sMOMENT models to measured in vivo data.

The AutoPACMEN model generator needs as main input the metabolic model as SBML file [26]. This SBML description must include gene-enzyme-reaction associations with standard (UniProt) enzyme identifiers. The model generator retrieves the molecular weights of proteins automatically from the UniProt protein database [25]. In addition, since the k_cat_ values are central for the enzyme constraints, AutoPACMEN includes a specifically engineered automatic k_cat_ retrieval method. AutoPACMEN can access the publicly available enzymatic databases SABIO-RK [17] and BRENDA [18]. Optionally, the user can also provide other (custom) k_cat_ database(s). Using the collected k_cat_ data from all these sources, AutoPACMEN chooses the k_cat_ values according to the number of entries for a reaction’s EC (Enzyme Commission) number as well as according to the substrates and the organism in which the k_cat_ values were measured. The substrate-depending k_cat_ search is supported using the BIGG database metabolite identifiers [27], while the organism-specific k_cat_ search uses NCBI TAXONOMY [28]. A full description of the approach to assign k_cat_ values to enzymes and reactions is described in the Additional file 1. In short, the k_cat_ selection algorithm works as follows: For each EC number of a reaction, k_cat_ values are collected from SABIO-RK and BRENDA. Then, for every reaction, its substrates and EC numbers are read out and standardized using BIGG identifiers. For every reaction’s EC number, the collected k_cat_ values are determined. Additionally, for every enzyme catalyzing the reaction, the optional custom k_cat_ values are retrieved, if available. Generally, from all these k_cat_ values, the ones measured with the reaction’s substrate and with enzymes from the metabolic model’s organism are preferred. If no value could be found for the given substrate and organism, then the values from the taxonomically nearest species are preferred. The constraints for the taxonomic distance are also relaxed if there are less than a minimum of 10 k_cat_ values for the given reaction. Finally, the mean value of all collected k_cat_ values is chosen. For all reactions for which no k_cat_ value could be found, a default k_cat_ representing the median of all found k_cat_ values is set.

Furthermore, if enzyme concentration measurements are given by the user, then AutoPACMEN includes explicit enzyme (concentration) variables in the model as explained in the Methods section.

The described AutoPACMEN model generator is written in Python 3 and requires a Python version > = 3.7. It can be used as console program or as Python module. Aside of Python’s standard library, the model generator also uses the modules biopython [29], cobrapy [23], click, openpyxl, pebble, requests and xlsxwriter.

The AutoPACMEN model calibrator consists of Python and MATLAB scripts and uses flux data to fit the enzyme pool variable *P* as well as the k_cat_ values both used in eq. (7) and (8). The objective function of these optimizations reads as follows.

13 $$ \underset{P,{k}_{cat}}{\mathit{\operatorname{Minimize}}}\ {\sum}_{growth\ scenarios\ j}{\sum}_{measured\ fluxes\ {v}_{ij}^m}{w}_{i,j}\max \left({v}_{ij}^m/{v}_{ij}^p,{v}_{ij}^p/{v}_{ij}^m\right) $$

where ${v}_{ij}^m$ is the measured flux of reaction *i* in scenario *j*, ${v}_{ij}^p$ the corresponding predicted flux and *w*_*ij*_ a weighting coefficient to optionally set preferences for certain measurements. This objective function ensures that the relative error of predicted vs. measured fluxes is minimized. The model calibrator makes use of MATLAB’s *fmincon* function, requires MATLAB version 2017a or higher and depends on the MATLAB metabolic modeling package *CellNetAnalyzer* [24, 30] which is used to make FBA predictions when calling *fmincon*. A separate Python script, which has the same dependencies as the AutoPACMEN model generator, is used for making a preselection of (sensitive) k_cat_ parameters for fitting (see Results and Additional file 1).

AutoPACMEN is free and open source under the Apache License. A GitHub repository has been created for AutoPACMEN (including a detailed manual and all scripts used to generate the sMOMENT-enhanced *i*JO1366* model): https://github.com/ARB-Lab/autopacmen

## Results

### sMOMENT and AutoPACMEN

As described in detail in the Methods section, we developed sMOMENT, a simplified formulation of the original MOMENT method for the integration of enzyme mass constraints in metabolic models. In contrast to MOMENT, sMOMENT requires much fewer variables than MOMENT and the enzyme constraints can be added as a minimal extension (one additional pseudo-metabolite and one additional pseudo-reaction) to the model’s stoichiometric matrix (Fig. 1). Thus, sMOMENT’s model representation not only reduces computational demand but also allows the use of standard software toolboxes for constraint-based modeling to analyze the resulting models.

In order to facilitate the construction of sMOMENT models, we developed AutoPACMEN (Automatic integration of Protein Allocation Constraints in Metabolic Networks). It consists of (1) the AutoPACMEN model generator for automatic generation of an sMOMENT-enhanced version of a stoichiometric metabolic model, and (2) the model calibrator which helps adjusting parameters of the included enzyme constraints based on measured data (Fig. 2).

The AutoPACMEN model generator can be used as console program or as Python module. The model calibrator can be used as MATLAB script using *CellNetAnalyzer* [24]. As primary input, the AutoPACMEN program reads the metabolic model from an SBML file [26]. The model generator can retrieve kinetic data from the proteomic databases SABIO-RK [17] and BRENDA [18] and optionally from a user-defined custom k_cat_ database (for further details see Methods section, Additional file 1 and AutoPACMEN’s user manual).

### The genome-scale *E. coli* model *i*JO1366 extended with sMOMENT

An exemplary run of AutoPACMEN was performed with the genome-scale *E. coli* model *i*JO1366 [19]. The SBML file of this model was provided as input. Since a large database of apparent enzyme turnover numbers (k_app_) was available in [31] these data were used as additional input to the k_cat_ values obtained from SABIO-RK and BRENDA resources. Note that k_app_ values reflect the actual (effective) turnover numbers as calculated from flux and proteomics data and may thus help to reduce overestimations from maximal turnover numbers (k_cat_).

A detailed step-by-step description and explanation of the AutoPACMEN run with *i*JO1366 can be found in Additional file 1 and in AutoPACMEN’s documentation. In the following, the sMOMENT-enhanced metabolic model of *i*JO1366 delivered by AutoPACMEN is denoted by *i*JO1366*. Compared to the parent model *i*JO1366 (Table 1), *i*JO1366* increased its number of reactions by 595 of which 594 simply arise due to the necessary splitting of enzymatically catalyzed reversible reactions into two irreversible (forward and backward) reactions representing the same metabolic capability. The true change in the behavior of the model stems from the integration of the protein pool pseudo-metabolite and of the pseudo reaction for synthesis of this metabolite with an upper limit determined by the maximum protein pool (see eq. (8) and Fig. 1). In total, AutoPACMEN could assign k_cat_ values to 1155 reactions of *i*JO1366, which goes far beyond the k_cat_ parametrization in the original MOMENT study (513 k_cat_ values including split reversible reactions).

**Table 1:** Model size of *i*JO1366 and *i*JO1366*

	*i*JO1366	*i*JO1366*
Number of reactions	2583	3178
Number of metabolites	1805	1806

### Fitting parameters of *i*JO1366*

Generally, enzyme-constrained models need model validation, i.e. some fitting to experimental data to (a) determine an appropriate upper limit for the protein pool *P* and (b) to adjust the original k_cat_ values to some extent to improve the agreement of model predictions with experimental data. As input for the parameter fitting of *i*JO1366* we used two sources, namely flux data (growth rate, substrate uptake and product excretion rates) for aerobic and anaerobic growth of *E. coli* K-12 MG1655 on glucose given in [32] as well as growth rates of *E. coli* exhibited on 23 additional substrates [13]. The latter dataset was also used in the original MOMENT paper for parameter fitting [13].

In a first step, we calibrated the protein pool variable *P* (needed as upper bound for *v*_*Pool*_ in eq. (8)) by fitting the predicted maximal growth rate for aerobic and anaerobic growth on glucose conditions against values reported in [32] and obtained a value of 0.095 g/gDW (for a detailed description of the calibration steps see also section 2.5 in Additional file 1). With this value, the iJO1366* predicts a maximal growth rate of 0.73 h^− 1^ for aerobic growth on glucose which matches exactly the value reported for *E. coli* K-12 MG1655 in [32]. It is important to notice that fitting parameter *P* with given flux data implicitly also accounts for averaged saturation effects: the maximum turnover number *k*_*cat*,*i*_ of a reaction *i* is often not reached in the cell due to saturation effects. The effective (or apparent) turnover number *k*_*app*,*i*_ is therefore typically lower and can be written as a saturation-corrected value of *k*_*cat*,*i*_: *k*_*app*,*i*_ = *σ*_*i*_ ∙ *k*_*cat*,*i*_ with 0 ≤ *σ*_*i*_ ≤ 1. Equation (7) then reads

14 $$ \sum {v}_i\bullet \frac{MW_i}{\sigma_i{k}_{cat,i}}\le P. $$

Since the *σ*_*i*_ are not known (and not fitted as independent variables), fitting the protein pool *P* in eq. (14) to reproduce given flux data then means that actually the *effective* protein pool ${P}_{eff}=\hat{\sigma}\bullet P$ is determined (where $\hat{\sigma}$ is the averaged saturation level) which is then used to bound *v*_*Pool*_ in eq. (8) (cf. also [11]). Only in the extreme case where all enzymes operate at maximum turnover (full saturation: $\hat{\sigma}=1$) we have *P*_*eff*_ = *P*.

The (effective) protein pool variable was fixed to the determined value of 0.095 g/gDW in all subsequent analyses. Next, in order to obtain realistic model behavior also for anaerobic growth on glucose, we manually identified four k_cat_ values of *i*JO1366* related to fermentation pathways that apparently required changes (see Additional file 1). For example, as found by AutoPACMEN, the EC number 1.2.1.10 of the acetaldehyde dehydrogenase (ACALD) for the direction with acetyl-CoA as educt is associated with a k_cat_ that is too low to achieve the high ethanol production rates of *E. coli* under anaerobic conditions (and there was no value in SABIO-RK with this educt). With more biological data (e.g., if k_app_ measurements were available for anaerobic conditions) this manual adjustment could be replaced with the automated workflow described in the following paragraph.

In a subsequent step we finally further optimized the k_cat_ values to improve the predictions with respect to the growth rates for 24 different substrates (glucose and the 23 other substrate-growth-rate pairs from [13]). Here we used AutoPACMEN’s model calibrator routines for fitting k_cat_ values (see Methods). As a preliminary step, the model calibrator identifies reactions whose k_cat_ value can be optimized for a growth rate prediction of one substrate without changing the results for other substrates. As a result of this selection process, only 96 out of the 1155 reactions with k_cat_ values were eventually selected for calibration. The adjusted k_cat_ values can be interpreted either as correction of the original k_cat_ values or as an adaptation of the k_cat_ values to the apparent turnover numbers (k_app_) under saturation levels of the respective growth conditions. The resulting model *i*JO1366* with the adapted k_cat_ values is provided in SBML format in Additional file 3 and was used for all further analyses described below.

### Growth-rate predictions of *i*JO1366*

**Fig. 3:**
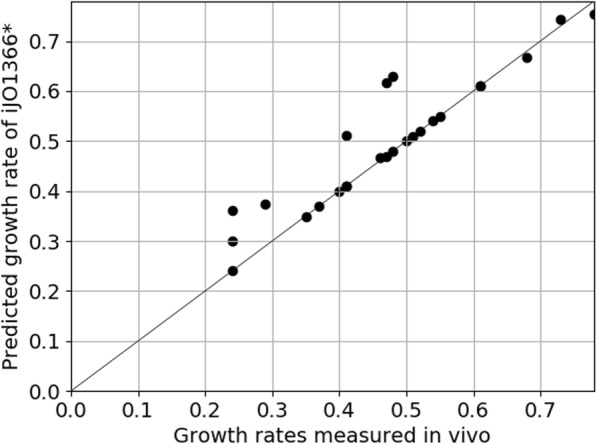
Scatter plot of *i*JO1366*-predicted and of measured in vivo growth rates for 25 different growth conditions. The in vivo data were taken from [13, 32] as described in the main text; more information can be obtained in Additional file 2. The black diagonal represents the identity function f(x) = x

Figure 3 shows the growth rate predictions of *i*JO1366* for 25 growth scenarios for which measurements were available ([13, 32]): 24 different substrates including glucose under aerobic as well as anaerobic growth (these scenarios were also used for the parameter fitting in the previous section). Importantly, no explicit flux bounds were set for the substrate uptake rates in these scenarios; substrate uptake is instead limited by the enzyme constraints.

The growth rate predictions of the fitted sMOMENT model correlate very well with the in vivo data with a Pearson correlation coefficient of 0.93 and a Spearman correlation coefficient of 0.91. The MOMENT version of *i*JO1366 applied to 24 of the 25 different growth rates yielded 0.47 for the Pearson as well as for the Spearman correlation coefficient [13].

### Prediction of exchange fluxes and of flux ranges

**Fig. 4:**
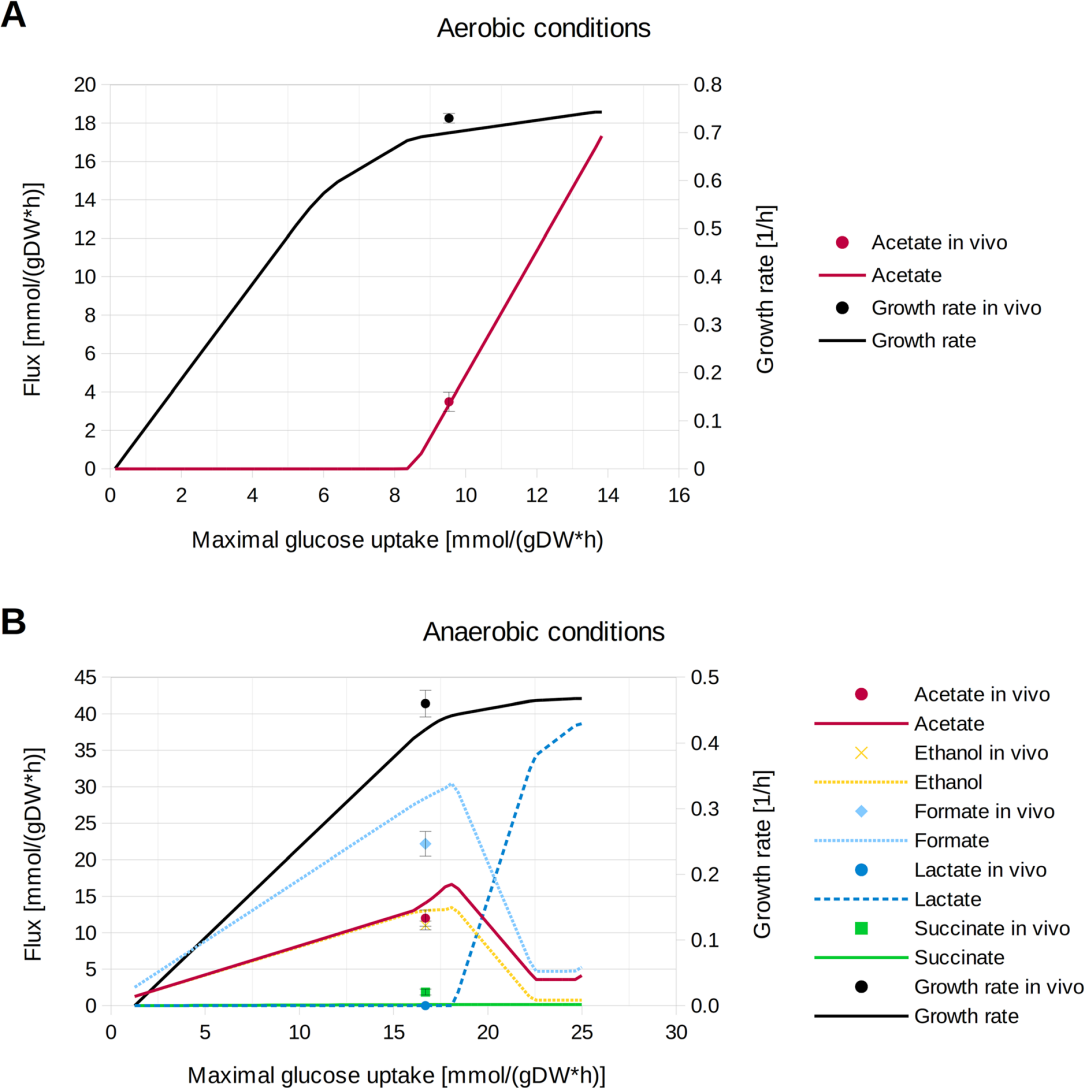
Predicted exchange fluxes of *i*JO1366* for the full range of all possible glucose uptake rates under (**a**) aerobic and (**b**) anaerobic conditions. Measured in vivo rates taken from [32] are also shown, together with their standard deviations (note that the (yellow) data point for the ethanol flux in (**b**) lies directly underneath the (red) data point of the acetate flux value; likewise the yellow line lies to a large extent directly underneath the red line). For a more detailed data set of this analysis see Additional file 2. An FVA shows that the exchange fluxes are unique for optimal growth at the respective substrate uptake rates

Going beyond maximal growth rate predictions shown in the previous section, we next intended to compare predicted vs. measured exchange fluxes (for substrate and major fermentation products) for aerobic and anaerobic growth on glucose (Fig. 4). Here, we assumed substrate-limited growth which limits the substrate uptake rate. We simulated the model with different glucose uptake rates ranging from the minimum (aerobic growth: 0.14 mmol/(gDW*h), anaerobic growth: 1.26 mmol/(gDW*h); these fluxes are needed for producing a minimum amount of ATP for maintenance metabolism) up to the maximum (aerobic: 13.83 mmol/(gDW*h), anaerobic: 24.99 mmol/(gDW*h)) of all possible substrate uptake rates in the model and determined for each uptake rate the resulting exchange fluxes when the growth rate is maximized. For aerobic conditions we found that the optimized model *i*JO1366* displays fully respiratory metabolism (without production of side products except CO_2_) until a critical glucose uptake rate is reached beyond which acetate excretion takes place. Thus, unlike *i*JO1366 and without adding further (e.g. oxygen uptake) constraints, *i*JO1366* can explain this well-known overflow metabolism of *E. coli* [10] solely by the inclusion of enzyme constraints. We also found a very good agreement of predicted rates for growth and acetate excretion with measured fluxes from [32] at a glucose uptake rate of 9.53 mmol/gDW/h.

Afterwards, we performed the same simulations for anaerobic growth with different glucose uptake rates. Consistent with biological knowledge, *i*JO1366* predicts a dominant excretion of ethanol, formate and acetate as fermentation products for a wide range of substrate uptake rates. The combined operation of these pathways gives the maximum (anaerobic) yield of 2.5 ATP per molecule glucose. For a substrate uptake rate of 16.69 mmol/(gDW*h) the predicted exchange fluxes agree again very well with measurements from [32]. Interestingly, *i*JO1366* predicts increasing lactate production rates (and reduced rates for all other fermentation products) for very high glucose uptake rates, however, the net gain in growth rate for this shift is only marginal and thus probably not relevant in vivo. However, in [33] it was shown that under conditions with large fluxes in the central metabolism, lactate synthesis might become the preferred fermentation pathway, possibly due to its reduced protein costs compared to the combined action of the ethanol, acetate and formate fermentation pathways.

**Fig. 5:**
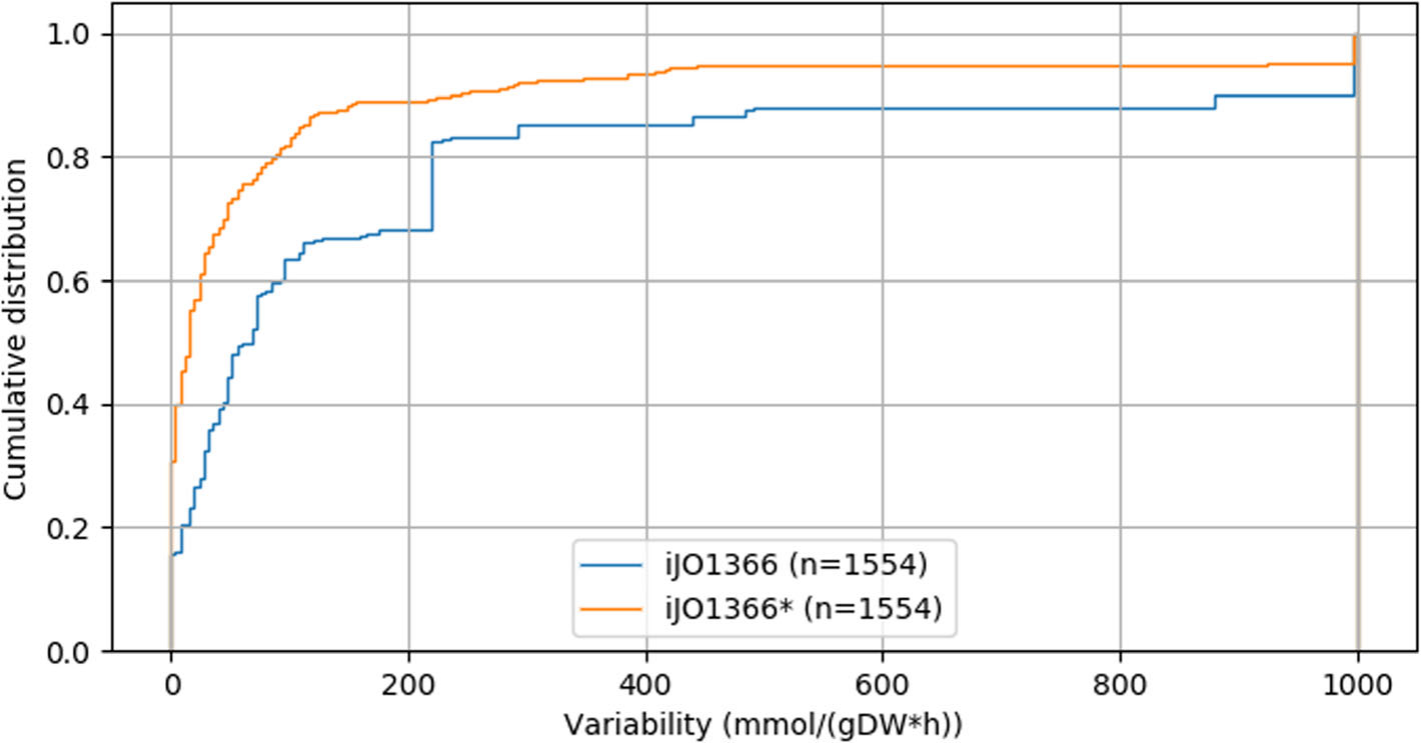
Comparative cumulative distributions of the flux variabilities of *i*JO1366 and *i*JO1366* (both with split reversible reactions) for aerobic growth with a maximum glucose uptake rate of 9.53 mmol/(gDW*h). For a detailed summary of the FVA results see Additional file 2. Reactions with zero flux (blocked reactions) were excluded

As further step to compare the solution spaces of the original *i*JO1366 and the sMOMENT-enhanced *i*JO1366* model, we performed flux variability analysis in both models for aerobic growth on glucose with a maximal glucose uptake rate of 9.53 mmol/(gDW*h) (corresponding to the measured value in [32]). In both models, all reversible reactions were split into two irreversible reactions and the exchange reactions for all carbon metabolites were inactivated except for the standard fermentation products acetate, ethanol, formate, succinate, lactate, and CO_2_ (a full list of the closed exchange reactions and of the flux variability analysis results can be found in the Additional file 2). As shown by the cumulative distribution in Fig. 5, *i*JO1366* has significantly reduced flux variabilities compared to *i*JO1366. Whereas 81 fluxes in *i*JO1366 are practically unbounded (reaching the artificial maximum bound of 1000) only 3 of those fluxes exist in *i*JO1366*. Moreover, every reaction in *i*JO1366* has either a reduced or identical flux range compared to *i*JO1366. These results highlight that the introduced enzyme constraints, consisting just of a single additional reaction and metabolite, significantly narrow down the flux space.

### Influence of enzyme constraints on metabolic engineering strategies

We used the minimal cut set (MCS) approach [7, 21, 34] to compute and compare metabolic engineering strategies in the *E. coli* genome-scale model with and without enzyme constraints. As application example we calculated MCSs with up to 6 reaction knockouts for the growth-coupled production of the commodity chemicals ethanol and succinate as well as for the amino acids leucine and valine in both *i*JO1366 (with split enzymatically catalyzed reversible reactions) and *i*JO1366*. The MCS were calculated for anaerobic conditions with the following constraints: the maximal glucose uptake rate in *i*JO1366 was set to 15 mmol/(gDW*h), while this rate was not explicitly bounded in *i*JO1366*. In both models, as in the FVA study, the exchange reactions for all carbon metabolites were disabled except for the standard fermentation products (acetate, ethanol, formate, succinate, lactate, CO_2_) and the respective target product. For each target product, a minimal growth rate of 0.1 h^− 1^ and a specific minimal product yield (1.4 mol/mol for ethanol, 1.0 mol/mol for succinate, 0.2 mol/mol for leucine and 0.3 mol/mol for valine) was demanded, irrespective of the growth rate (strong coupling [35]). The MATLAB script for enumerating the MCSs with *CellNetAnalyzer* [30, 36] can be found in AutoPACMEN’s distribution.

The complete results of the MCS computations can be found in Additional file 2. Table 2 summarizes the results indicating very heterogeneous MCS distributions between the two models. Interestingly, for ethanol as target product we found that protein allocation constraints in *i*JO1366* cause a significantly higher number of metabolic engineering strategies (58% more MCS in *i*JO1366* compared to *i*JO1366). A closer look at the interrelationships of the MCSs reveals that approximately a quarter of the 7168 MCS in *i*JO1366* are shared with *i*JO1366 while the largest fraction (~ 60%) represents MCS with knockout strategies that do not exist (also not as superset or subset of computed MCS) in *i*JO1366. Especially interesting is the fact that there are 231 MCS in *i*JO1366* that are (proper) subsets of (1516) MCS in *i*JO1366. The reduced number of required interventions in these MCS indicate that ethanol secretion is already enforced to a certain extent by the enzyme constraints. On the other hand, a few of such cases also exist in the other direction where (11) MCS of *i*JO1366 are subsets of (101) MCS in *i*JO1366*. Similar results are obtained for succinate as target product, although the fraction of identical MCS in both models is larger.

**Table 2:** Comparative results of the minimal cut sets found for different target products in *i*JO1366 and *i*JO1366*. The given rounded percentages of subset and superset categories refer to the respective total number of minimal cut sets. The complete results can be found in Additional file 2

Product	Ethanol		Succinate		Leucine		Valine	
Model	*i*JO1366	*i*JO1366*	*i*JO1366	*i*JO1366*	*i*JO1366	*i*JO1366*	*i*JO1366	*i*JO1366*
#MCS	4538	7168	7801	9619	196	0	29,290	3712
#MCS up to size 3	0	0	0	0	0	0	0	0
#MCS of size 4	87	189	135	215	24	0	240	0
#MCS of size 5	678	871	1918	2148	32	0	3100	48
#MCS of size 6	3773	6108	5748	7196	140	0	25,950	3664
#MCS being subset of the other model’s MCS	11 (0.2%)	231 (3.2%)	21 (0.3%)	174 (1.8%)	0	0	0	0
#MCS being superset of the other model’s MCS	1516 (33.4%)	101 (1.4%)	1218 (15.6%)	42 (0.4%)	0	0	0	0
#MCS shared by both models	1899 (41.8%)	1899 (26.5%)	6141 (78.7%)	6141 (63.9%)	0	0	3280 (11.2%)	3280 (88.4%)
#MCS neither identical, superset or subset of the other model’s MCS	1112 (24.6%)	4937 (68.9%)	421 (5.4%)	3262 (33.9%)	196 (100%)	0	26,010 (88.8%)	432 (11.6%)

A different picture is seen for the amino acids leucine and valine. First, not a single MCS is found for leucine in *i*JO1366* while at least 196 could be computed for *i*JO1366. Here it is to be expected that pathways for leucine synthesis enforced by MCS in *i*JO1366 are not valid in *i*JO1366* due to some limitation by the enzyme costs. Using FBA we found that it is generally possible to reach the given leucine yield in the iJO1366* under the given minimal growth rate, however, coupling cannot be enforced, at least not with up to 6 knockouts. In the case of valine, the number of MCS (3712) in *i*JO1366* is relatively high but markedly reduced compared to *i*JO1366 (29290). Importantly, while 3664 MCS are identical in both models, not a single MCS that exists only in either model is a reduced version (subset) of the other indicating that also rather different strategies arise in both models. The results of the MCS study thus demonstrate that the application of sMOMENT may lead to new biotechnological metabolic engineering strategies which would not have been found without enzyme allocation constraints.

## Discussion

In this work we presented three major developments. First, we introduced the sMOMENT method for simplified inclusion of (enzymatic) protein allocation constraints in metabolic models. We then developed the AutoPACMEN toolbox allowing automatic construction and calibration of sMOMENT models. Finally, we applied AutoPACMEN to construct the enzyme-constrained version *i*JO1366* of the genome-scale *E. coli* model *i*JO1366 and compared these two models demonstrating how the added enzyme allocation constraints affect major model properties and predictions.

MOMENT [13], a further development of FBAwMC [12], was one of the first constraint-based modeling approaches accounting for enzyme mass constraints by integrating enzyme-specific (kinetic and molecular weight) parameters. sMOMENT introduced herein is based on the same approach but uses a simplified and standardized representation of the constraints. There are three key differences to MOMENT: (i) sMOMENT does not require explicit variables for enzyme concentrations. (ii) sMOMENT simplifies the treatment of isozymes catalyzing the same reaction by considering the most conservative constraint (i.e., the enzyme with the lowest costs in terms of required protein mass). This does not change the results of simulations. (iii) The enzyme constraints are integrated in a compact manner (addition of just one pseudo metabolite and one pseudo reaction) in the standard formulation of constraint-based metabolic models which enables their analysis and simulation with dedicated tools as well as their storage and export as SBML model.

A related method to MOMENT and sMOMENT is GECKO [11] where the metabolic enzymes as well as their formation and usage are explicitly included in the metabolic model as species and reactions, respectively, together with the overall enzyme mass constraints. One major motivation for this explicit representation in GECKO was the possibility to directly integrate measured enzyme concentrations which can further constrain the model. However, this comes to the price that the models can become very large. For example, the fully expanded GECKO model for *i*JO1366 (generated with AutoPACMEN where all enzymes were given some (pseudo-)concentration measurements) contains 7728 reactions and 4166 metabolites, which is an enormous increase compared to 3178 reactions and 1806 metabolites in the sMOMENT model *i*JO1366* (cf. Table 1). Computationally expensive analyses (such as the enumeration of minimal cut sets) become hard or even impossible in such a huge network. We also compared the flux predictions of the raw *i*JO1366* (before adjusting the k_cat_ values with the model calibrations) with the respective GECKO version of the *i*JO1366 model (with the same maximal protein pool value of 0.095 g/gDW) and did not find any differences if no protein measurements are provided. Furthermore, although not used herein, we described in the Methods section how given enzyme concentration measurements can be properly included during the automated construction of an sMOMENT model while still keeping the model as small as possible. As mentioned above and described in the Methods section, a fully expanded GECKO model can also be generated with AutoPACMEN if needed.

As for MOMENT and GECKO, sMOMENT models focus on protein mass constraints and are therefore simpler than the more advanced resource balance analysis (RBA [14];) and Metabolism and Expression (ME) models [16] where all steps of gene expression (e.g., transcription and translation) and other processes are explicitly included. These models have increased predictive capabilities but lead to very complex models with a large number of additional parameters (e.g., transcription efficiencies) which are often not known. Especially for organisms with few experimental data, sMOMENT, together with the AutoPACMEN toolbox, provides a first and relatively simple step towards inclusion of biosynthetic costs in constraint-based models.

The AutoPACMEN toolbox is, to our knowledge, the first program suite providing a virtually fully automated workflow for the integration and calibration of enzyme constraints in a given stoichiometric metabolic model. No such comprehensive toolbox was available for MOMENT whereas a set of manually editable and partly automated scripts were provided for generating GECKO models [11]. This GECKO toolbox allows retrieval of reaction-specific k_cat_ data, but only from the BRENDA database and it does not include the capability to automatically calibrate k_cat_ values. Furthermore, the Python scripts seem not be compatible with current versions of Python 3.

Another related toolbox was recently published for (semi-)automated construction of RBA models (RBApy [37]). As explained above, RBA needs a considerable amount of additional parameters. However, while parameter estimation via experimental data is supported by RBApy, automatic retrieval of many parameters (such as k_app_ values) from external databases is not possible.

Since the model generator of AutoPACMEN can be used either as console program or as Python modules, it can be easily integrated with other metabolic modeling programs. As the program suite depends on cobrapy [23], it can be already seen as an extension for it. The applicability of AutoPACMEN was demonstrated by the generation of the *i*JO1366* model, however, AutoPACMEN is ready to be used with any other constraint-based metabolic model (with standardized name space and gene-enzyme-reaction associations), regardless of the species they represent.

The calibrated enzyme-constrained genome-scale model for *E. coli, i*JO1366*, constructed herein with AutoPACMEN, is provided in SBML format in Additional file 3 and holds significant potential for diverse applications. *i*JO1366* is, to the best of our knowledge, the *E. coli* genome-scale model based on (simple) enzyme constraints with the widest coverage of k_cat_ values. If enzyme concentration measurements are available, AutoPACMEN can be used to integrate them, with minimal model extensions, in *i*JO1366*. Furthermore, by relaxing the protein pool variable *P* to a very high value, *i*JO1366* behaves as the original model *i*JO1366 thus allowing simultaneous simulation of *E. coli*’s metabolism with and without enzyme constraints.

The basic analyses conducted herein with *i*JO1366* already revealed interesting properties and several key differences to the original model *i*JO1366. The explanation and predictions of phenomena such as overflow metabolism with enzyme constraints is not new [10, 11, 38], however, it demonstrated the validity of *i*JO1366* under the given conditions. Moreover, the phenomenon of increased lactate synthesis under anaerobic conditions with high substrate uptake rates could be predicted. Furthermore, the conducted analysis of intervention strategies for different target products is the most comprehensive done so far for enzyme-constraint models and revealed important insights. In particular, while some strategies might be valid in both models, a significantly altered spectrum of minimal cut sets may result when enzyme constraints are included and enforcement of growth-coupled product synthesis may become easier (less interventions required) or harder (more interventions required). It thus seems worth to rigorously include enzyme constraints for computational strain design in metabolic engineering.

While enzyme-constrained models may exhibit a higher predictive and explanatory power than classical constraint-based models, they require as additional input three different types of enzyme parameters (protein pool *P*, k_cat_ values and the molecular weight of the enzymes). While the molecular weights can often be determined accurately, the k_cat_ values retrieved from the databases usually have a much higher uncertainty. They are difficult to measure (often only in vitro and not in vivo) and reported measurements sometimes differ by orders of magnitudes. Moreover, specific k_cat_ values are often not available for the organism under study and must then be taken from related species. Calibration of the original k_cat_ values and estimating the protein pool *P* from available flux measurements is thus essential to obtain meaningful predictions of enzyme-constrained models and is supported by AutoPACMEN. Moreover, AutoPACMEN also provides options to use different modes of k_cat_ value assignment (e.g., selection of a random or of the median or mean value from the relevant kcat values found in the databases) which can then be used to test the effect of different k_cat_ distributions on the model predictions.

## Conclusion

The methodological and tool developments presented herein pave the way for a simplified and routine construction and analysis of enzyme-constrained metabolic models. Moreover, the generated *i*JO1366* model allows exploration of the genome-scale metabolism of *E. coli* under enzyme mass constraints. First analyses of *i*JO1366* revealed several interesting properties and differences compared to the *i*JO1366 model emphasizing the importance of consideration of enzyme constraints in metabolic models.

## Supplementary information

**Additional file 1.** Additional information on construction of the *i*JO1366* model with the AutoPACMEN toolbox.

**Additional file 2.** List of disabled exchange reactions in all analyses with *i*JO1366*. Growth rate predictions. Exchange flux predictions. Flux variability analysis results. Minimal cut sets results.

**Additional file 3.** Final sMOMENT-enhanced and calibrated *i*JO1366 model (*i*JO1366*) in SBML format.

## Data Availability

A GitHub repository for AutoPACMEN (including a detailed manual, the cached database information from the data retrieval for *i*JO1366* and all scripts used for the generation of the sMOMENT-enhanced *i*JO1366 model) is available at: https://github.com/ARB-Lab/autopacmen Project name: AutoPACMEN Project home page: https://github.com/ARB-Lab/autopacmen Operating system(s): Cross-platform Programming language: Python, Matlab Other requirements: biopython, cobra, click, openpyxl, pebble, requests, xlsxwriter, CellNetAnalyzer License: Apache License, Version 2 Any restrictions to use by non-academics: none.

## Abbreviations

FBA: Flux Balance Analysis
FBAwMC: Flux Balance Analysis with Molecular Crowding
FVA: Flux Variability Analysis
GECKO: Genome-scale model enhancement with Enzymatic Constraints, accounting for Kinetic and Omics data
MCS: Minimal Cut Sets
MOMENT: MetabOlic Modeling with ENzyme kineTics
RBA: Resource Balance Analysis
sMOMENT: short MOMENT
