# Supplementary material for: Automatic construction of metabolic models with enzyme constraints
Source: BMC Bioinformatics. 2020 Jan 14;21:19. doi: 10.1186/s12859-019-3329-9 (PMC6961255; doi:10.1186/s12859-019-3329-9)
Supplement: Supplementary file 1 — Additional file 1. Additional information on construction of the iJO1366* model with the AutoPACMEN toolbox. [file 12859_2019_3329_MOESM1_ESM.pdf]

Supplementary File 1

Automatic Construction of Metabolic Models with Enzyme Constraints

Pavlos Stephanos Bekiaris[1] and Steffen Klamt[1]

[1]Max Planck Institute for Dynamics of Complex Technical Systems, Magdeburg, Sandtorstr. 1, Germany

Table of Contents

1 Detailed explanation of k_{cat} value selection by AutoPACMEN....................2
1.1 k_{cat} databases and datasets....................2
1.2 General problems of k_{cat} retrieval and assignments....................2
1.3 AutoPACMEN's approach of retrieving and selecting k_{cat} values....................3
2 Generation and calibration of the enzyme-constrained *E. coli* model iJO1366* using AutoPACMEN....................6
2.1 Introduction....................6
2.2 Preparation of metabolic model and external data sources....................6
2.3 Preparation of growth scenarios....................6
2.4 Model generator run....................7
2.5 Model calibration....................8
2.5.1 Protein pool calibration....................8
2.5.2 Manual k_{cat} adjustment....................9
2.5.3 Automated k_{cat} calibration....................10
3 References....................10

A detailed description of the AutoPACMEN toolbox can be found in AutoPACMEN's manual which is provided as part of the AutoPACMEN package. Herein we only (1) describe the specific rules of AutoPACMEN for extracting and assigning kcat values (possibly from different resources) and (2) provide a step-by-step description of how the sMOMENT-enhanced model iJO1366* was generated with the AutoPACMEN toolbox.

1 Detailed explanation of k_{cat} value selection by AutoPACMEN

1.1 k_{cat} databases and datasets

Two major public k_{cat} databases are BRENDA (1) and SABIO-RK (2). In both databases, k_{cat} values can be sorted according to the EC number of the represented reaction. Additionally, for each k_{cat} value, the respective educts (and direction) of a given reaction as well as the organism from which the analyzed enzyme comes from are usually specified in these databases. Apart from BRENDA and SABIO-RK, AutoPACMEN also allows consideration of other (user-defined) k_{cat} values (or of measured apparent turnover numbers k_{app}), for example, from literature references where these values were directly or indirectly determined (3). We refer to these additional data as (external) custom database.

1.2 General problems of k_{cat} retrieval and assignments

While the molecular weight of an enzyme can be quite simply determined from its amino acid sequence, the determination of an enzyme's k_{cat} for a specific reaction is much harder and includes a much higher degree of uncertainty. This has multiple reasons:

1. k_{cat} values are often determined *in vitro*, whose experimental conditions may not be representative for the enzyme's physiological conditions *in vivo*.
2. In most k_{cat}-determining experiments, heterologously expressed recombinant enzymes are used. This can e.g. lead to differences in the enzyme's folding and adds an additional level of uncertainty to the measured k_{cat}.
3. For some types of reactions, k_{cat} values are not given for all possible substrates. One common approach to handle this is to take a mean value of k_{cat} values of this type of reaction with any kind of substrate. Since enzymes can have a high substrate specificity, this mean value does not have to be a necessarily realistic.
4. It is also possible that there is no k_{cat} for a given enzyme and organism. One way to handle this is to select k_{cat} values from the taxonomically nearest organisms, since the role and amino acid sequence of enzymes is more likely to be comparable within more related organisms than within less related ones.
5. Finally, for some reactions and reaction classes, no k_{cat} are available at all. In these cases, k_{cat} values of similar reactions (e.g. reactions with similar EC numbers) may be taken with the trade-off of a higher uncertainty.

1.3 AutoPACMEN's approach of retrieving and selecting k_{cat} values

AutoPACMEN's model generator retrieves and assigns k_{cat} values thereby seeking to address the 5 problems mentioned in the previous chapter. The k_{car} retrieval method consists of two parts:

1. Preparation of data sources

AutoPACMEN can process data from the k_{cat} databases BRENDA and SABIO-RK, as well as from a user-created custom database with protein-dependent k_{cat} data as explained above. In addition, the extraction and assignment process uses the BIGG database's definition of metabolite identifiers and their names (4).

The k_{cat} data of BRENDA and SABIO-RK is processed into machine-readable JSON (JavaScript Object Notation) files by AutoPACMEN (for an explanation on how to use the programs themselves, see next section and AutoPACMEN's manual). These JSON files are structured in the following way: For each EC number, a list of possible substrate names is given. The substrate names are stored in their BIGG identifier form (obtained from the annotations of the SBML version of the metabolic model) in order to be able to combine the data from BRENDA and SABIO-RK. If a substrate name could not be associated with a BIGG identifier, it is stored with the general substrate name "REST". For each of these substrate names, a list of organism names of the analyzed enzymes with the given substrate is given. Finally, for each organism, the list of associated k_{cat} values is given.

The optional custom k_{cat} database has to be prepared in the form of a JSON, too, whose structure is as follows: Its main keys are the name of each included protein (the protein name has to be the same as the one in the metabolic model). Each protein name has two associated necessary fields. One field is "kcats" which includes the list of k_{cat} values that were determined for this protein. The other field is "direction", which includes the names of all protein-associated reactions as keys, and "reverse" or "forward" as associated values in order to indicate in which reaction direction the protein-dependent k_{cat} was determined.

2. Selection of k_{cat} values

Once the k_{cat} data from SABIO-RK, BRENDA, and the optional custom database have been stored as JSON, the actual selection process of k_{cat} values for each reaction of a metabolic model can start. In the following description of the k_{cat} selection algorithm (algorithm 1), the combined k_{cat} database from SABIO-RK and BRENDA is called the "SABIO-RK/BRENDA database":

Algorithm 1: k_{cat} selection algorithm of AutoPACMEN.

Step A: For each (reversible or irreversible) reaction of the metabolic model, do the following

I. Retrieve all EC numbers of this reaction.

II. Delete all EC numbers which include a wild-card symbol such as "*" or "-". For example, the EC number "1.1.1.-" would be deleted. This is done in order to prevent the inclusion of k_{cat} values from dissimilar reactions.

III. Get the educts for each the forward and reverse direction of the reaction in the form of

BIGG identifiers using the downloaded BIGG metabolites text file.

IV. For each the forward and reverse direction of the reaction, continue with step B1 if no EC numbers are remaining, otherwise continue with step B2.

Step B1: For the current reaction and current direction do

I. Get the list of all enzymes which are given in the reaction’s gene rules of the metabolic model.

II. For each enzyme, check if there is an entry in the custom database and in the right reaction direction. If no such entry can be found, proceed with step C. Otherwise, retrieve all k_{cat} values from the fitting enzyme, and finally select the maximal k_{cat} value of the retrieved k_{cat} values.

III. Finally, find the minimal maximal value of all enzymes from which k_{cat} values from the custom database and assign this value to the reaction’s k_{cat}.

IV. Continue with the next reaction, starting from step A.

Step B2: For the current reaction and current direction do

I. Find all SABIO-RK and BRENDA database k_{cat} entries for all of the reaction’s EC numbers. These entries contain the measured k_{cat} value of the enzyme as well as the organism and the substrate of the of the measurement experiment.

II. Check if the BIGG identifiers of the educts and/or products can be found in any of the found SABIO-RK/BRENDA k_{cat} entries.

III. If II turns out to be true, get the entries with the fitting educts (for the forward direction) or products (for the reverse direction) only. If substep II turns out to be false, get all entries, regardless of the substrate. Merge all resulting selected entries into one combined meta-entry.

IV. For each organism of the k_{cat} entries in the meta-entry which was selected in substep III, get the taxonomic distance from the metabolic network’s organism to the k_{cat} entry’s organism.

V. Continue with step B3.

Step B3: Using the list of taxonomic distances in the selected meta-entry, do the following:

I. Start with the minimal taxonomic distance

II. For each k_{cat} entry of the meta-entry, get all k_{cat} values from all organisms up to the currently selected minimal taxonomic distances using NCBI TAXONOMY (5).

III. If the list of collected k_{cat} values is shorter than 10, go to the next taxonomic distance and redo substep II.

IV. Perform step B1. If the optional custom k_{cat} database includes a protein-specific entry for at least one of the current reaction’s associated enzymes, add it to the list of k_{cat} values.

V. Take the mean of all selected k_{cat} values and select it as k_{cat} for the reaction and its currently analyzed direction. If no k_{cat} value could be found for this enzyme for any organism go to

Step C.

VI. Continue with the next reaction in Step A

Step C: (If no k_{cat} could be found for the reaction)

Assign a default k_{cat} value for this reaction which is the median of all k_{cat} values used for all of the model's reaction with a selected k_{cat} .

After this algorithm has been finished, a reaction-k_{cat} database with k_{cat} values for each direction of the metabolic model's is created. This database is a JSON text file and has the following form (where names starting with $ stand for a variable name, e.g. $REACTION_ID stands for any of the included reactions IDs):

```
{
    "$REACTION_ID": {
        "forward": $kcat_value_forward,
        "reverse": $kcat_value_reverse
    },
    (...)
}
```

2 Generation and calibration of the enzyme-constrained *E. coli* model iJO1366* using AutoPACMEN

2.1 Introduction

In the following we outline how the AutoPACMEN Model Generator and Model Calibrator were used with the *E. coli* K-12 model iJO1366 (6) in order to obtain the calibrated enzymeconstrained model iJO1366*. iJO1366 is a widely used a genome-scale model with 2583 reactions and 1805 metabolites, for both of which it uses BIGG identifiers. Additionally, as required, reactions include gene rules and EC-number annotations.

This exemplary run is delivered as a script which is included in the AutoPACMEN package. Its program run and custom analysis scripts start with "ec_model_2019_06_25". All downloadable external data was retrieved on the 25th June 2019. The non-script project files – i.e., inputs and outputs – can be found in the subfolders starting with the same name.

2.2 Preparation of metabolic model and external data sources

The iJO1366 model was downloaded from the BIGG database's website (URL: http://bigg.ucsd.edu/data_access, accessed on September 9, 2019) in SBML format and lies in the subfolder "ec_model_2019_06_25_input".

The BRENDA database was downloaded as text file from https://www.brenda-enzymes.org/download_brenda_without_registration.php (accessed on September 9, 2019) and stored in the subfolder "ec_model_2019_06_25_input" as "brenda_download.txt" (the latter file is not included in AutoPACMEN's distribution due to its large file size). The k_{cat} values of SABIO-RK are received on the fly (using its API). For the custom k_{cat} database we use here values from (3). In order to obtain the custom k_{cat} database data as a AutoPACMEN-compatible JSON file, a custom Python script (called "ec_model_2019_06_25_data_read_supplementary_table_of_keff_paper.py") was written in order to convert the data from the supplementary table S2 of (3) into a protein-dependent k_{cat} JSON called "gene_id_data_mapping.json" in this run's subfolder "ec_model_2019_06_25_input_keff_paper".

A dataset of the BIGG metabolite definitions was downloaded as text file from http://bigg.ucsd.edu/data_access (accessed on September 9, 2019) and stored in the subfolder "ec_model_2019_06_25_input" as "bigg_models_metabolites.txt".

2.3 Preparation of growth scenarios

23 *in vivo* growth rates for different substrates were taken from (7), excluding the growth rate value for glucose. For glucose as substrate, the aerobic and anaerobic *in vivo* growth rate values of (8) were taken (using the values with the *E. coli* strain K-12 MG1655). Additionally, the secretion values from (8) were used for the manual calibration step (see chapter 2.5.2).

Additionally, a "standard exchange scenario" was defined for the iJO1366: In the original iJO1366, many exchange reactions are open and it is possible that a secretion of metabolites occurs even if there is no biological indication for the possibility under the viewed circumstances. Therefore, all exchanges of carbon metabolites except for standard fermentation products are disabled (flux is zero) in this "standard exchange scenario" (see also Supplementary File 2).

2.4 Model generator run

For this run, the AutoPACMEN Model Generator was used as Python library. All used Python steps are included in the main script "ec_model_2019_06_25_sMOMENT_iJO_CREATION.py" in AutoPACMEN's main folder, which is intersected in the following steps:

Step 1. Parses the BIGG metabolites text file "bigg_models_metabolites.txt" into the more easily machine-readable "psb_orth_bigg_id_name_mapping.json" in the subfolder "ec_model_2019_06_25_output".

Step 2. Parses the BRENDA database text file "brenda_download.txt" into the more easily machine-readable k_{cat} JSON "kcat_database_brenda.json" in the subfolder "ec_model_2019_06_25_output".

Step 3. Reads out all EC numbers of the iJO1366 model and creates a new k_{cat} JSON named "kcat_database_brenda_for_model.json" which includes all k_{cat} entries for the EC numbers, either with or without wildcard search (in the current AutoPACMEN implementation, k_{cat} entries are only used if they resulted from a search without wildcards, i.e., if a k_{cat} value for a reaction was found by the reaction's actual EC number). This JSON is in the subfolder "ec_model_2019_06_25_output".

Step 4. Using the SABIO-RK API, all EC numbers of iJO1366 are searched (on-the-fly) in SABIO-RK. The resulting k_{cat} entries for every EC number – again resulting from a search with or without wildcards – are stored as "kcat_database_sabio_rk.json" in the subfolder "ec_model_2019_06_25_output".

Step 5. Combines the BRENDA and SABIO-RK k_{cat} databases into the JSON "kcat_database_combined.json", preferring entries which did not result from a wildcard search in the subfolder "ec_model_2019_06_25_output".

Step 6. Creates the reaction $\leftrightarrow k_{cat}$ mapping for iJO1366. Its k_{cat} entry sources are "kcat_database_combined.json" and "bigg_id_data_mapping.json", stored in the subfolder "ec_model_2019_06_25_output".

Step 7. Creates two relevant XLSX spreadsheets in the subfolder "ec_model_2019_06_25_output" which are used later in step 9. The 1st of these spreadsheets is "psb_orth_protein_data.xlsx" which includes two worksheets: In the first worksheet, the user can set the protein pool *P*. By default, this value is at 0.095 mmol/(gDW*h). In the second worksheet, the user can set optional enzyme concentration data. Since no such data is used for iJO1366*'s generation, no concentration data was written inside. The 2nd spreadsheet is "psb_orth_enzyme_stoichiometries.xlsx". In this spreadsheet, the user can enter internal stoichiometries for complexes or the number of used single enzymes per

reaction step. As no such stoichiometry data was used for the generation of iJO1366*, this spreadsheet remained unchanged.

Step 8. Creates the protein↔molecular weight mapping for each protein contained in iJO1366’s gene rules, using the UniProt API. The resulting JSON is called “psb_orth_protein_id_mass_mapping.json” and is located in the subfolder “ec_model_2019_06_25_output”.

Step 9. The final sMOMENT model generation script. The resulting (raw) enzyme-constraint model is saved as “iJO1366_sMOMENT_2019_06_25.xml” in the subfolder “ec_model_2019_06_25_output”. Importantly, otherwise included enzyme allocation constraints were disabled for the gas-exchanging reactions CO2text, O2tex and H2tex as there is no biological justification for the assumption that carbon dioxide, oxygen and hydrogen need porine enzymes for membrane exchanges.

Step 10. This step loads “iJO1366_sMOMENT_2019_06_25.xml” from the previous step and assigns the flux bounds of exchange reactions according to the “standard exchange scenario” (see section 2.4). The glucose uptake was unconstrained. The resulting model, which we call here iJO1366*A is saved as “iJO1366_sMOMENT_2019_06_25_STANDARD_EXCHANGES_SCENARIO.xml” in the subfolder “ec_model_2019_06_25_output”.

2.5 Model calibration

2.5.1 Protein pool calibration

To obtain a valid enzyme-constrained metabolic model, in the next step a calibration of the (effective) protein pool *P* and afterwards of the k_{cat} values needed to be performed (see main document). Calibration of the protein pool variable *P* was manually done with iJO1366*A by fitting the predicted maximal growth rate with glucose under aerobic and anaerobic conditions against known values from (8). Here, we did not use the AutoPACMEN Model Calibrator as only two growth rates were used for fitting. The manually determined optimal *P* value was 0.095 g/gDW (see Table S1 for the resulting growth rates), which is used in the next optimization steps. We call the model with this protein pool iJO1366*B.

*Table S1: Comparison of protein-pool-optimized iJO1366*B model (with standard exchange scenario) with* in vivo *data from (8). The chosen maximal protein pool value is 0.095 g/gDW.*

**Condition**	**Protein pool optimized iJO1366*B** Maximal growth rate [h^{-1}]	**Monk et al., 2016** (8) Measured growth rate and standard deviation [h^{-1}]
Glucose, aerobic	0.73	0.73 ± 0.01
Glucose, anaerobic	0.51	0.46 ± 0.02

2.5.2 Manual k_{cat} adjustment

In the model iJO1366*B, obtained in the previous step, the secretion of acetate and glucose uptake rates for aerobic conditions were already similar to *in vivo* values (8) under aerobic conditions (see Table 2 for the aerobic secretion values of the final optimized model).

However, under anaerobic conditions, there a larger discrepancy in the secretion of fermentation products between the model and the *in vivo* data could be observed (see Table S2). The most apparent discrepancy is a high lactate secretion in the iJO1366*B model.

To improve this behavior under anaerobic conditions, we introduced very few (just 4) minimal changes of k_{cat} values of the following reactions/enzymes:

- The k_{cat} value of PFL (pyruvate formate lyase) was 10 times increased.
- The k_{cat} value of ACALD_reverse (acetaldehyde dehydrogenase (acetylating)) was 5 times increased. The original AutoPACMEN-selected k_{cat} value was 8.82 s^{-1} which is apparently too small to achieve high ethanol production rates observed under anaerobic conditions with *E. coli*.
- The k_{cat} value of ALCD2x_reverse (Alcohol dehydrogenase (ethanol)) was 10 times decreased.
- The k_{cat} value of LDH_D_reverse (D-lactate dehydrogenase) was 10 times decreased.

We denote the resulting model iJO1366*C. The script with which the manual optimization is applied on the model is “ec_model_2019_06_25_modeling_apply_manual_changes.py”, the manually changed model is called “iJO1366_sMOMENT_2019_06_25_STANDARD_EXCHANGES_SCENARIO_MANUAL_CHANGES.xml” and is located in the subfolder “ec_model_2019_06_25_output_optimization”.

*Tab. 2 Anaerobic organic compound secretion rates of iJO1366*B and of iJO1366*C obtained after manually optimization of four* k_{cat} *values, compared to* in vivo *values of (8)*

**Product**	**Monk et al., 2016 (8)** *in vivo anaerobic* secretion rates [mmol/(gDW*h)] at glucose uptake rate of 16.69 mmol/(gDW*h)	**sMOMENT model iJO1366*B** Anaerobic secretion rates [mmol/(gDW*h)] at glucose uptake rate of 16.69 mmol/(gDW*h)	**Optimized sMOMENT model iJO1366*C** Anaerobic secretion rates [mmol/(gDW*h)] at glucose uptake rate of 16.69 mmol/(gDW*h)
Ethanol	11.22 ± 0.6	2.23	13.3
Formate	22.17 ± 1.69	6.14	28.6
Acetate	11.71 ± 1.14	2.42	13.5
Lactate	0 ± 0	23.2	0
Succinate	1.86 ± 0.4	0.12	0.141

2.5.3 Automated k_{cat} calibration

Using the iJO1366*C model (see the previous chapter), an automatic k_{cat} optimization was performed in order to get better growth rate predictions for the 25 growth scenarios. The optimization was performed using AutoPACMEN's Model Calibrator. The actual run of the calibrator for this model optimization can be found in the MATLAB script "optimization_run_fmincon_2019_06_25.m" in the subfolder "AutoPACMEN_Model_Calibrator_MATLAB". The scenarios used for the optimization are in the described in the JSON "optimization_scenarios.json" in the same subfolder.

In an initial step, using the Python script "ec_model_2019_06_25_CALIBRATOR_get_reaction_flux_control_and_differential_reactions.py" , we first determined all reactions whose k_{cat} value deletion has an impact on the growth rate on exactly one out of the 25 (substrate) scenario. The k_{cat} values of only those reactions were then be adjusted in the subsequent optimization step.

A total of 96 reactions were selected for calibration (the list of these reactions can be found in the mentioned MATLAB script). Each of the scenarios had at least 2 affected reactions.

For the automatic optimization procedure a maximal k_{cat} change factor of 50 was used. The optimization run itself took around 1 day and stopped after the default MATLAB Optimization Toolbox reached its limit of 3000 objective function evaluations per fmincon optimization. The resulting k_{cat} values were integrated in the Python script "ec_model_2019_06_25_optimization_apply_fmincon_optimized_kcats.py", which converted the iJO1366*C model into the final iJO1366* model by adapting the resulting k_{cat} values.

The resulting final optimized model is called "iJO1366star.xml" and located in the subfolder "AutoPACMEN_Model_Calibrator_MATLAB". In addition, it is given as Additional File 3 with an opened glucose exchange reaction.

3 References

1. Jeske L, Placzek S, Schomburg I, Chang A, Schomburg D. BRENDA in 2019: a European ELIXIR core data resource. Nucleic Acids Res. 2019 Jan 8;47(D1):D542–9.

2. Wittig U, Kania R, Golebiewski M, Rey M, Shi L, Jong L, et al. SABIO-RK—database for biochemical reaction kinetics. Nucleic Acids Res. 2012 Jan 1;40(D1):D790–6.

3. Valgepea K, Adamberg K, Seiman A, Vilu R. Escherichia coli achieves faster growth by increasing catalytic and translation rates of proteins. Molecular BioSystems. 2013;9(9):2344–58.

4. King ZA, Lu J, Dräger A, Miller P, Federowicz S, Lerman JA, et al. BiGG Models: A platform for integrating, standardizing and sharing genome-scale models. Nucleic Acids Res. 2016 Jan 4;44(D1):D515–22.

5. Federhen S. The NCBI Taxonomy database. Nucleic Acids Res. 2012 Jan 1;40(D1):D136–43.

6. Orth JD, Conrad TM, Na J, Lerman JA, Nam H, Feist AM, et al. A comprehensive genome-scale reconstruction of Escherichia coli metabolism—2011. Molecular Systems Biology. 2011 Jan 1;7(1):535.

7. Adadi R, Volkmer B, Milo R, Heinemann M, Shlomi T. Prediction of Microbial Growth Rate versus Biomass Yield by a Metabolic Network with Kinetic Parameters. PLOS Computational Biology. 2012 Jul 5;8(7):e1002575.

8. Monk JM, Koza A, Campodonico MA, Machado D, Seoane JM, Palsson BO, et al. Multi-omics Quantification of Species Variation of Escherichia coli Links Molecular Features with Strain Phenotypes. Cell Systems. 2016 Sep 28;3(3):238-251.e12.
